# Prognostic value of elevated lipoprotein (a) in patients with acute coronary syndromes: a systematic review and meta-analysis

**DOI:** 10.3389/fcvm.2024.1362893

**Published:** 2024-05-09

**Authors:** Guochun Wang, Maoyin Xia, Cai Liang, Feng Pu, Sitai Liu, Dongxia Jia

**Affiliations:** ^1^ The Clinical College of North Sichuan Medical College, Nanchong, Sichuan, China; ^2^ Department of General Practice, Sichuan Mianyang 404 Hospital, The Second Affiliated Hospital of North Sichuan Medical College, Mianyang, Sichuan, China

**Keywords:** acute coronary syndromes, lipoprotein (a), prognosis, outcome, mortality

## Abstract

**Background:**

Elevated lipoprotein (a) level was recognized as an independent risk factor for significant adverse cardiovascular events in acute coronary syndrome (ACS) patients. Despite this recognition, the consensus in the literature regarding the prognostic significance of elevated lipoprotein (a) in ACS was also limited. Consequently, we conducted a thorough systematic review and meta-analysis to evaluate the prognostic relevance of elevated lipoprotein (a) level in individuals diagnosed with ACS.

**Methods and results:**

A thorough literature review was conducted by systematically searching PubMed, Embase, and Cochrane databases until September 2023. This review specifically examined cohort studies exploring the prognostic implications of elevated lipoprotein (a) level in relation to major adverse cardiovascular events (MACE), including death, stroke, non-fatal myocardial infarction (MI), and coronary revascularization, in patients with ACS. The meta-analysis utilized aggregated multivariable hazard ratios (HR) and their respective 95% confidence intervals (CI) to evaluate prognostic implications between high and low lipoprotein (a) levels [the cut-off of high lipoprotein (a) level varies from 12.5 to 60 mg/dl]. Among 18,168 patients in the identified studies, elevated lipoprotein (a) was independently associated with increased MACE risk (HR 1.26; 95% CI: 1.17–1.35, *P* < 0.00001) and all-cause mortality (HR 1.36; 95% CI: 1.05–1.76, *P *= 0.02) in ACS patients. In summary, elevated lipoprotein (a) levels independently forecast MACE and all-cause mortality in ACS patients. Assessing lipoprotein (a) levels appears promising for risk stratification in ACS, offering valuable insights for tailoring secondary prevention strategies.

**Systematic Review Registration:**

PROSPERO (CRD42023476543).

## Introduction

Acute coronary syndromes (ACS) encompasses ST-segment elevation myocardial infarction (STEMI), non-ST-segment elevation myocardial infarction (NSTEMI), and unstable angina (UA) (1–4). They are common in older adults (5)^,^ and yet the incident has increased in younger people (6, 7). Annually, over 7 million individuals worldwide receive ACS diagnoses (8, 9). Roughly 5% succumb before hospital discharge (2, 8–10). Subsequent ACS survivors frequently face major adverse cardiovascular events (MACE), which encompass recurrent ischemic incidents and mortality (11–13). Despite advancements in medical therapy, the one-year incidence of MACE post-ACS has risen to 9.2% (14). Identifying a predictive indicator for MACE after ACS is imperative for improved prognostic outcomes. Recently, lipoprotein(a) [Lp (a)] has emerged as an independent risk factor linked to MACE following ACS (15–19). Nevertheless, conflicting evidence surrounds the prognostic significance of elevated blood Lp (a) levels in ACS patients. For instance, the FORTIAM investigation (20), a multicenter cohort study in Spain involving 1,371 acute myocardial infarction (AMI) patients across 15 hospitals, revealed a poorer prognosis in those with elevated Lp (a) levels at admission. Consistent findings were reported by Andrea Kallmeyer et al. and Si-qi Yang et al. (21, 22). Conversely, a Vietnam-based observational cohort study (23) yielded disparate results, suggesting no correlation between Lp (a) levels ≥ 50 mg/dl during AMI and MACE or all-cause mortality. No comprehensive systematic review or meta-analysis has yet assessed the prognostic significance of elevated Lp (a) levels in ACS patients regarding MACE and all-cause mortality. This meta-analysis aims to investigate the prognostic relevance of baseline blood lipoprotein(a) levels in predicting MACE and all-cause mortality among ACS patients.

## Methods

The present evidence-based analysis adheres to the guidelines stipulated in the 2020 PRISMA (Preferred Reporting Items for Systematic Reviews and Meta-Analysis) statement (24). The study protocol was prospectively registered in the PROSPERO database under the registration number CRD42023476543. The comprehensive PRISMA 2020 checklist is provided in Supplementary Table S1. Our systematic literature review employed a comprehensive search strategy, utilizing PubMed, Embase, and Cochrane databases, with the search scope extending until September 2023. We specifically targeted English-language studies exploring the incidence of MACE and/or all-cause mortality in ACS patients. MACE were defined as all-cause mortality, stroke, non-fatal MI, and coronary revascularization. The comparison focused on individuals with high levels of Lp (a) vs. those with low Lp (a) levels. We searched the databases using the following terms: “Lipoprotein(a)”, “Lipoprotein Lp (a) “, “Lipoprotein a”, “Acute Coronary Syndrome”, “Coronary Syndrome, Acute”, “Syndrome, Acute Coronary”, “Syndromes, Acute Coronary”, “Myocardial Infarction”, “Infarction, Myocardial”, “Cardiovascular Stroke”, “Stroke, Cardiovascular”, “Myocardial Infarct”, “Infarcts, Myocardial”, “Heart Attack”, “Angina, Unstable”, “Unstable Anginas”, “Angina Pectoris, Unstable”, “Unstable Angina Pectori”, “Unstable Angina”, “Angina at Rest”, “Angina, Preinfarction”, “Preinfarction Angina”, “Myocardial Preinfarction Syndrome”, “Preinfarction Syndrome, Myocardial”, and “Syndromes, Myocardial Preinfarction” et al. We searched PubMed with the following detailed search strategy: ((((“Angina, Unstable"[Mesh]) OR (((((((((((((((((((Angina, Unstable) OR (Anginas, Unstable)) OR (Unstable Anginas)) OR (Angina Pectoris, Unstable)) OR (Angina Pectori, Unstable)) OR (Unstable Angina Pectori)) OR (Unstable Angina Pectoris)) OR (Unstable Angina)) OR (Angina at Rest)) OR (Angina, Preinfarction)) OR (Anginas, Preinfarction)) OR (Preinfarction Angina)) OR (Preinfarction Anginas)) OR (Myocardial Preinfarction Syndrome)) OR (Myocardial Preinfarction Syndromes)) OR (Preinfarction Syndrome, Myocardial)) OR (Preinfarction Syndromes, Myocardial)) OR (Syndrome, Myocardial Preinfarction)) OR (Syndromes, Myocardial Preinfarction))) OR ((“Myocardial Infarction"[Mesh]) OR ((((((((((((((Myocardial Infarction) OR (Infarction, Myocardial)) OR (Infarctions, Myocardial)) OR (Myocardial Infarctions)) OR (Cardiovascular Stroke)) OR (Cardiovascular Strokes)) OR (Stroke, Cardiovascular)) OR (Strokes, Cardiovascular)) OR (Myocardial Infarct)) OR (Infarct, Myocardial)) OR (Infarcts, Myocardial)) OR (Myocardial Infarcts)) OR (Heart Attack)) OR (Heart Attacks)))) OR ((“Acute Coronary Syndrome"[Mesh]) OR ((((((Acute Coronary Syndrome) OR (Acute Coronary Syndromes)) OR (Coronary Syndrome, Acute)) OR (Coronary Syndromes, Acute)) OR (Syndrome, Acute Coronary)) OR (Syndromes, Acute Coronary)))) AND (((((“Lipoprotein(a) “) OR (“ Lipoprotein Lp (a) “)) OR (“Lipoprotein (a) “)) OR (“Lipoprotein a “)) OR (“Lipoprotein (a-)”)). The exhaustive search methodology is outlined in Supplementary Table S2. Additionally, a thorough manual review of reference lists from all eligible studies was undertaken. Two investigators independently performed the search and assessment of included studies. Any discrepancies in the literature search were resolved through consensus after careful deliberation.

### Identification of eligible studies

Eligible studies met the following criteria: (1) utilization of randomized controlled, cohort, or case–control designs; (2) examination of adults with diagnosed ACS; (3) primary focus on comparing MACE and/or all-cause mortality between ACS patients with high and low Lp (a) levels; (4) assessment of at least one MACE (e.g., death, stroke, non-fatal MI, or coronary revascularization) with concurrent evaluation of all-cause mortality; and (5) availability of sufficient data to compute the Hazard Ratio (HR). Exclusions comprised reviews, letters, editorial comments, case reports, conference abstracts, pediatric articles, unpublished works, and non-English publications.

According to ACS criteria, the syndrome comprises UA, NSTEMI, and STEMI. Therefore, studies that focused on stable angina, chronic coronary artery disease (CAD) were also excluded.

### Data extraction

Two independent investigators (G.C.W and M.Y.X) performed data extraction. Discrepancies were resolved through intervention by a third investigator (S.T.L or D.X.J) to establish consensus. Extracted data from the studies included primary author, publication year, study duration, location, design, ACS types, sample size, follow-up duration, participant age, body mass index (BMI), high-density lipoprotein cholesterol (HDL-c), low-density lipoprotein cholesterol (LDL-c) levels, history of prior medical therapy, diabetes prevalence, Lp (a) threshold, MACE, and mortality-related outcomes. For studies reporting continuous variables as median with range or interquartile range, a validated mathematical approach was employed to derive mean and standard deviation (21–28).

### Quality assessment

Study quality was assessed using the Newcastle–Ottawa Scale (NOS) (29), categorizing studies scoring seven to nine points as high quality (30). Two independent reviewers evaluated evidence quality in eligible studies, resolving discrepancies through consensus discussions.

### Statistical analysis

Evidence synthesis was performed using Review Manager version 5.4.1 (Cochrane Collaboration, Oxford, UK). Survival variables were assessed using Hazard Ratios (HR), and metrics were presented with corresponding 95% confidence intervals (CIs). Heterogeneity assessment utilized the inconsistency index (*I*^2^) (31), where *I*^2^ values exceeding 50% indicate significant heterogeneity. In instances of significant heterogeneity (*I*^2^ > 50%), a random-effects model estimated combined HR; otherwise, a fixed-effect model was applied (31). Furthermore, we conducted one-way sensitivity analyses to assess the influence of included studies on combined outcomes showing substantial heterogeneity. Publication bias was evaluated visually using funnel plots generated with Review Manager 5.4.1 (Cochrane Collaboration, Oxford, UK) and statistically through Egger's regression tests (32) implemented with Stata version 15.0 (Stata Corp, College Station, TX, USA) for outcomes with 10 or more included studies. A *P*-value < 0.05 indicated statistically significant publication bias.

## Results

### Literature search and study characteristics

**Figure 1:**
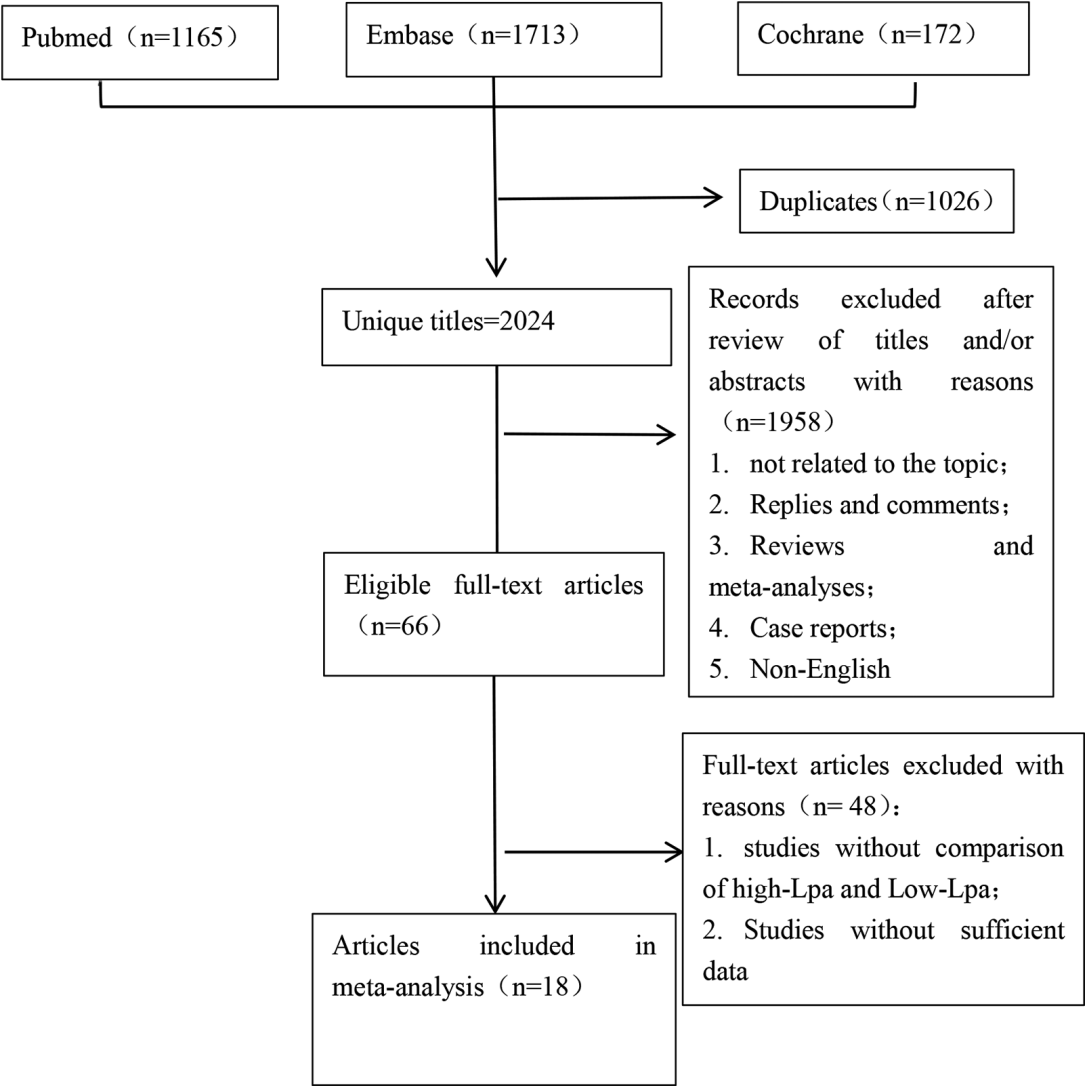
Flowchart of the systematic search and selection process.

The schematic representation of the methodical search and selection procedure is depicted in Figure 1. A comprehensive literature search across PubMed (*n* = 1,165), Embase (*n* = 1,713), and Cochrane (*n* = 172) databases identified a total of 3,050 pertinent articles. Following the elimination of duplicate publications, a meticulous review of titles and abstracts resulted in the inclusion of 2024 articles for further consideration. Ultimately, 18 full-text articles, encompassing a total of 18,168 patients (13,843 male, 4,325 female), were selected for the comprehensive pooled analysis (20–23, 25–28, 33–42). Of these articles, 5 were prospective cohort studies (21, 23, 34, 35, 40), 10 were retrospective cohort studies (20, 22, 25, 26, 28, 33, 36–39), and 3 were observational cohorts (4, 27, 41). The encompassed investigations in this analysis spanned the period from 2009 to 2023. The sample sizes exhibited considerable variability, ranging from 88 to 2007 participants, resulting in a cumulative cohort of 18,168 patients. The duration of follow-up varied notably, extending from 2.93 to 66 months. It is noteworthy that the defined cutoff value for Lp (a) demonstrated heterogeneity across the studies incorporated in this review. Table 1 comprehensively outlines the distinctive characteristics, level of evidence, and quality assessments of each included study. The median (range) quality score, indicative of methodological rigor, was determined to be 7 (5–9). Notably, 14 studies were acknowledged for their high quality, indicating the strength of their methodologies and findings within the scope of this comprehensive analysis (21, 23, 26–28, 33–40, 42). The quality assessment details of all eligible are presented in Supplementary Table S3.

**Table 1:** Baseline characteristics of include studies and quality assessment.

Author	Study period	Area	Study design	Multi-/single center	Follow up period	Gender	
						Male	Female
Kallmeyer et al. (21)	2006–2016	Spain	Prospective	Multicenter	62.76	805	237
Hoang et al. (23)	2020	Viet Nam	Prospective	Single center	2.93	128	71
Xue et al. (34)	2015–2020	China	Prospective	single center	30	1080	279
Park et al. (35)	2011–2018	Korea	Prospective	Single center	35.88	1,346	562
Zhu et al. (36)	2017–2020	China	Retrospective	Single center	36	350	166
Yang et al. (22)	2014–2020	China	Retrospective	Multicenter	17	574	191
Wang et al. (33)	2012–2019	China	Retrospective	Single center	30	1,850	468
Dai et al. (37)	2015–2018	Japan	Retrospective	Single center	55.2	196	66
Takahashi et al. (38)	2008–2017	Japan	Retrospective	Single center	26.4	876	255
Sang et al. (25)	2011–2020	China	Retrospective	Single center	66	335	201
Cui et al. (39)	2017–2019	China	Retrospective	Multicenter	16	593	211
Mitsuda et al. (26)	2010–2012	Japan	Retrospective	Single center	36	131	45
Roth et al. (27)	2004–2012	Austria	Observational	Single center	38.4	939	306
Gómez et al. (20)	–	Spain	Retrospective	Multicenter	6	1,150	221
Mitsuda et al. (28)	2007–2014	Japan	Retrospective	Single center	36	538	130
Gencer et al. (40)	2009–2012	Switzerland	Prospective	Multicenter	12	1,391	320
Li et al. (41)	2015–2019	China	Observational	Single center	46.8	919	387
Wohlfahrt et al. (42)	2017–2020	czech republic	Observational	Single Center	19	642	209

Author	No. of patients	Age	BMI	HDL-c	LDL-c
Kallmeyer et al. (21)	1,042	60.7 ± 3.3	27.9 ± 1.0	38.3 ± 2.5	118.7 ± 36.9
Hoang et al. (23)	199	62.9 ± 2.9	22.9 ± 0.7	36.0 ± 2.2(*n* = 192)	120.4 ± 9.9(*n* = 192)
Xue et al. (34)	1,359	63.4 ± 12.6	24.0 ± 3.6	NA	112.1 ± 34.8
Park et al. (35)	1,908	65.2 ± 12.8	24.0 ± 3.4	NA	106.1 ± 39.8
Zhu et al. (36)	516	66.0 ± 9.8	24.3 ± 3.1	41.8 ± 10.4	96.7 ± 36.3
Yang 2022 (22)	765	65.67 ± 3.40	NA	44.5 ± 12.4	100.5 ± 35.6
Wang et al. (33)	2,318	58.8 ± 11.9	25.7 (23.6–28.0)	42.9 (35.6-9.1)	108.7 ± 36.5
Dai et al. (37)	262	67.5 ± 13.3	NA	49.7 ± 14.0	120.0 ± 43.9
Takahashi et al. (38)	1,131	68.2 ± 11.9	24.0 ± 3.9	48.2 ± 13.6	124.0 ± 36.6
Sang et al. (25)	536	81 (80–83)	24.39 ± 3.21	42.1 ± 2.5	85.8 ± 5.8
Cui et al. (39)	804	66 ± 13	NA	46.0 (36.7–58.4) 44.0 (36.0–47.2) 45.2 (32.1,53.0)	88.5 (56.83,119.1) 104.8 (83.9,126.8) 106.3 (86.6,142.3)
Mitsuda et al. (26)	176	65.4 ± 12.6	23.9 ± 1.1	44.0 (38.3–53.8) 46.0 (40.0–52.0)	105.0 (88.3–128.0) 124.0 (100.0–145.0)
Roth et al. (27)	1,245	60.3 ± 2.9	28 (25–31) 27 (25–31) 27 (25–30) 27 (24–31)	43 (37–50) 42 (36–50) 43 (35–51) 44 (37–51)	115 (88–146) 130 (99–158) 128 (93–155) 136 (107–164)
Gómez et al. (20)	1,371	57.4 ± 10.7	27.4 ± 4.3	41 ± 12	138 ± 38
Mitsuda et al. (28)	668	65.8 ± 11.7	24.0 ± 0.7	43.0 ± 1.8	115.0 ± 6.4
Gencer et al. (40)	1,711	63.9 ± 12.6	27.1 ± 4.4	46.4 ± 15.5	131.4 ± 42.5
Li 2022 (41)	1,306	66 ± 13.0	25.2 ± 3.6	37.9 ± 11.2	102.4 ± 36.7
Wohlfahrt et al. (42)	851	65.1 ± 11.9	28.8 ± 4.9	43.3 ± 12.8	11.3 ± 42.9

Author	MACE (outcome)		All-cause mortality (outcome)		Lp (a) threshold (mg/dl)	Quality score
	HR (95%CI)	*P*	HR (95%CI)	*P*		
Kallmeyer et al. (21)	1.44 (1.02–2.03)	0.036	NA	NA	60	7
Hoang et al. (23)	NA	NA	0.50 (0.20–1.80)	NA	50	6
Xue et al. (34)	NA	NA	1.43 (1.21–1.68)	NA	19.1	7
Park et al. (35)	1.17 (0.87–1.57)	0.306	NA	NA	50	7
Zhu et al. (36)	1.63 (1.12–2.38)	0.012	NA	NA	30	8
Yang et al. (22)	2.07 (1.366–3.132)	<0.01	1.37 (0.737–2.550)	0.32	30	8
Wang et al. (33)	1.37 (0.98–1.92)	0.063	NA	NA	28.7	7
Dai et al. (37)	2.84 (1.25–6.60)	0.013	NA	NA	32	7
Takahashi et al. (38)	1.66 (1.05–2.61)	0.03	NA	NA	15	7
Sang et al. (25)	1.35 (1.024–1.790)	0.033	1.80 (1.286–2.532)	≤0.001	30	6
Cui et al. (39)	2.01 (1.476–3.784)	<0.001	NA	NA	30	8
Mitsuda et al. (26)	1.03 (1.011–1.048)	0.002	NA	NA	16.5	7
Roth et al. (27)	NA	NA	1.2 (0.7–1.9)	0.55	60	8
Gómez et al. (20)	1.65 (1.15–2.35)	NA	NA	NA	60	6
Mitsuda et al. (28)	1.02 (1.009–1.025)	<0.001	NA	NA	50	8
Gencer et al. (40)	1.05 (0.64-1.73)	0.84	0.82 (0.39-1.73)	NA	30	9
Li et al. (41)	2.79 (2.08–3.75)	<0.001	NA	NA	50	5
Wohlfahrt et al. (42)	NA	NA	2.92 (1.16–7.37)	0.023	12.5	8

### Impact of elevated lipoprotein (a) on major adverse cardiovascular events

**Figure 2:**
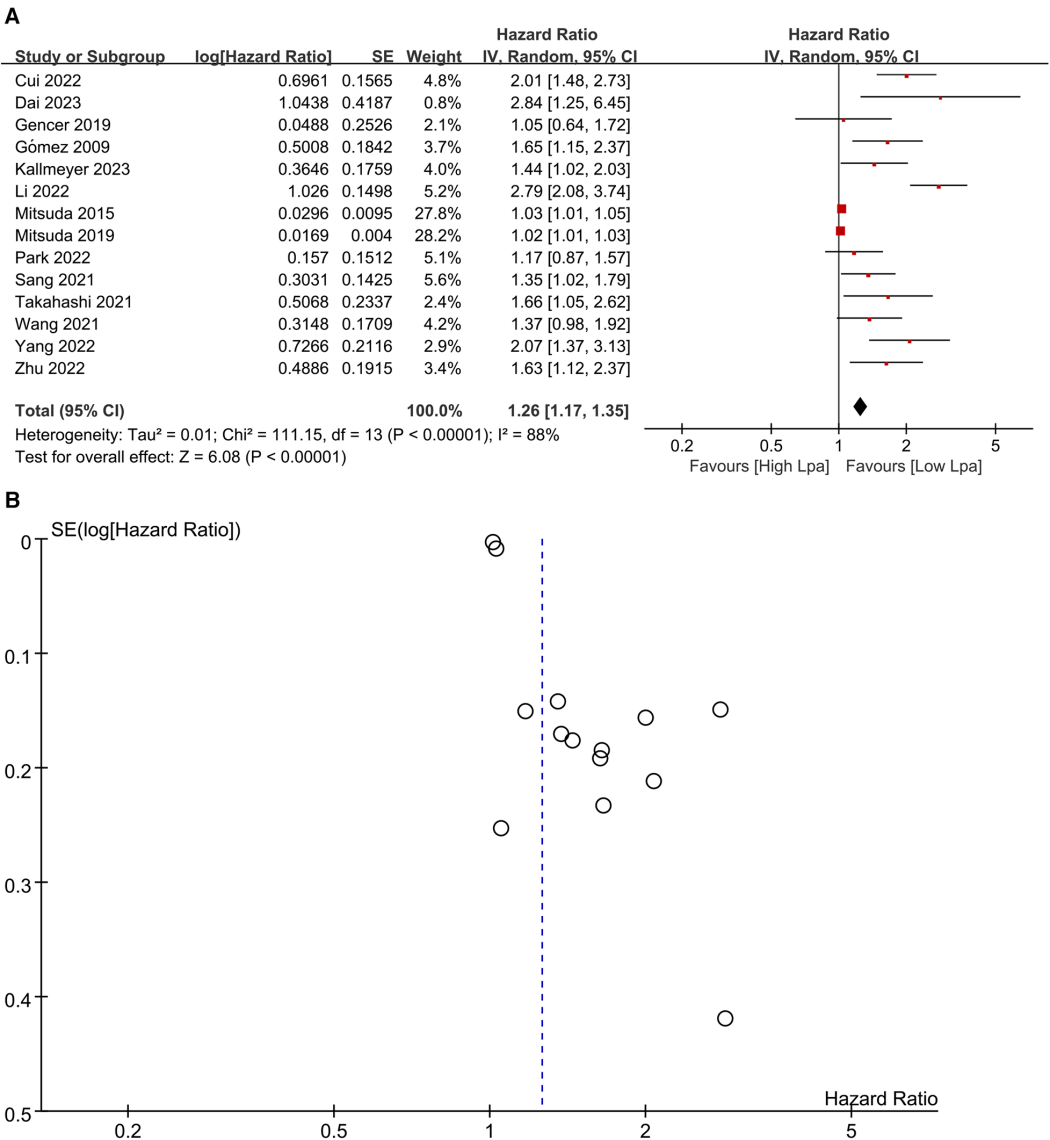
(**A**) Forest plots of major adverse cardiovascular events outcomes. (**B**) Funnel plots of major adverse cardiovascular events outcomes.

Fourteen studies (20–23, 25, 26, 28, 33, 36–41) investigated the prognostic significance of elevated Lp (a) levels in relation to MACE (Figure 2A). The meta-analysis utilizing a random-effects model revealed a pooled HR of 1.26 (95% CI: 1.17–1.35) for high compared to low category of Lp (a) levels, demonstrating a statistically significant association. However, notable heterogeneity was observed (*I*^2^ = 88%; *P *< 0.00001). A visual inspection of the funnel plot did not reveal any significant publication bias (Figure 2B). Nevertheless, Egger's test yielded a statistically significant result (*P* < 0.0001). Sensitivity analyses were performed by excluding two studies, both conducted by the same author (26, 28). The removal of these studies resulted in a discernible impact on the original pooled effect sizes of MACE, suggesting that the observed heterogeneity may be attributed to these specific studies.

### Impact of elevated lipoprotein (a) on all-cause mortality

**Figure 3:**
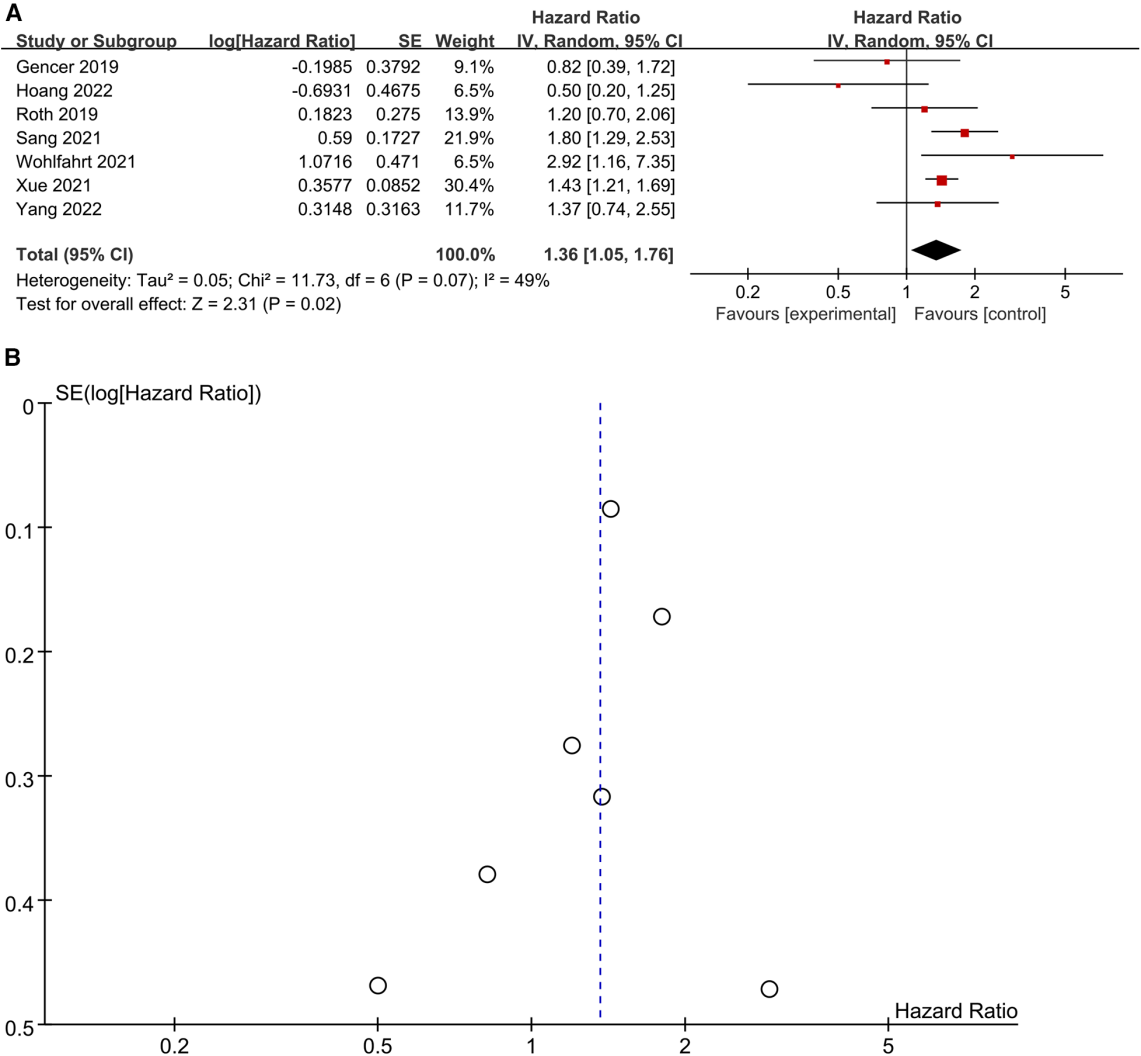
(**A**) Forest plots of all-cause mortality outcomes. (**B**) Funnel plots of all-cause mortality outcomes.

Seven studies (22, 23, 25, 27, 33, 34, 40) were included in the analysis to investigate the relationship between elevated Lp (a) levels and the risk of all-cause mortality (Figure 3A). Utilizing a random-effects model, the pooled Hazard Ratio (HR) for all-cause mortality was 1.36 (95% CI: 1.05–1.76) when comparing high to low category of Lp (a) levels. There was no significant heterogeneity observed (*I*^2^ = 49%; *P* = 0.02). Examination of the funnel plot revealed no significant publication bias (Figure 3B), and Egger's test did not show statistical significance (*P* = 0.518). Sensitivity analyses, involving the removal of each study one at a time, demonstrated minimal alterations to the original pooled effect sizes for all-cause mortality. This supported the stability of the results. In summary, the comprehensive analysis of these seven studies indicates a statistically significant association between elevated Lp (a) levels and an increased risk of all-cause mortality. The findings are robust, as evidenced by the lack of publication bias, low heterogeneity, and consistent results in sensitivity analyses.

### Subgroup analysis

In addition, we conducted a subgroup analysis to assess the correlation between elevated Lp (a) levels and the occurrence of MACE, as well as all-cause mortality specifically within the subgroup of patients diagnosed with ACS. We divided the patients into two subgroups according to the study design (prospective cohort vs. retrospective cohort), follow-up duration (≥3 years vs. < 3 years), Lp (a) level (Lp (a) > 30 mg/dl vs. Lp (a) threshold ≤30 mg/dl), area (Asia vs. Europe), and divided into 3 subgroups according to the types of ACS (ACS vs. AMI vs. STEMI). The findings from the subgroup analysis reveal a strong correlation between elevated Lp (a) levels and an increased risk of MACE across all subgroups, with the exception of the STEMI subgroup (HR 1.02, 95% CI: 1.00–1.04, *P *= 0.02), but the difference of all-cause mortality was not statistically significant in subgroup with prospective cohort (HR 0.94 95%CI: 0.50–1.78, *P *= 0.86), follow-up duration < 3 years (HR 1.23 95%CI: 0.82–1.86, *P *= 0.32), Europe area (HR 1.34 95%CI: 0.72–2.49, *P *= 0.36), and Lp (a) threshold > 30 mg/dl (HR 0.84 95%CI: 0.36–1.95, *P *= 0.69).

## Discussion

ACS stands as a predominant contributor to global morbidity and mortality, acting as a primary catalyst for coronary heart disease-associated hospitalizations and fatalities, thereby imposing a substantial disease burden (43). Lp (a), comprising apolipoprotein (a), apolipoprotein B-100, and lipid components like cholesterol, phospholipids, and triglycerides (44), has been identified as a promoter of atherosclerosis, inflammation, and thrombosis, emerging as an independent risk factor for ACS (45, 46). The 2019 ESC/EAS guidelines for dyslipidemia management advocate for at least one Lp (a) level assessment for every adult during their lifetime (47). The determination of Lp (a) levels may aid in identifying patients necessitating more intensive therapeutic interventions in ACS. Future investigations are imperative to discern whether reducing Lp (a) levels can confer cardiovascular benefits to ACS patients. The precise mechanisms driving Lp (a)'s predictive value in ACS patients remain enigmatic. Plausibly, Lp (a) exhibits dual pathogenicity, manifesting proinflammatory and antifibrinolytic effects (48). An alternative rationale suggests elevated Lp (a) may compromise endothelial and anticoagulant functions by promoting endothelial dysfunction and increasing phospholipid oxidation (49, 50). Ongoing research delves into the potential role of Lp (a) in risk stratification and residual risk modification (51), prompting the present meta-analysis.The primary findings of this meta-analysis underscore that an elevated Lp (a) level serves as an independent predictor of MACE and all-cause mortality in ACS patients. Individuals with high Lp (a) levels exhibited approximately 26% and 36% higher risks of MACE (HR 1.26, 95% CI: 1.17–1.35, *P* < 0.00001) and all-cause mortality (HR 1.36, 95% CI: 1.05–1.76, *P* = 0.02), respectively, compared to those with low Lp (a) levels. Notably, the findings in subgroup with STEMI for MACE were not significantly different in this paper, which may be due to the shorter follow-up periods in studies with STEMI. In addition, most of the studies about STEMI were large-scale and retrospective. Furthermore, the inclusion criteria varied slightly according to different studies (e.g., a Japan-based study in 2019 (28) included patients with index STEMI, while others did not make the declaration). On the other hand, the different results in subgroup analysis for all-cause mortality suggested that the prognostic value of Lp (a) level for all-cause mortality is associated with the study design, follow-up duration, and patients’ races (from different areas and with various Lp (a) levels). It is presumed that the results in subgroup analysis did not show statistical significance, maybe due to the smaller samples with longer follow-up durations (*n* = 4,885 for <3 years vs. *n* = 1,475 for ≥3 years), which suggests that the prognostic value of high Lp (a) levels for all-cause mortality may not be present in the short term. The differences in Lp (a) thresholds among various areas may result from genetic racial differences (52). All of which may lead to the differences among studies. The 2019 ESC/EAS guidelines and a statement from the American Heart Association suggest that adults should assess Lp (a) concentration once in lifetime, preferably in initial lipid panel test (47, 53). It is necessary to explain Lp (a) test results in the context of other risks and absolute global cardiovascular risk for ACS patients, and emphasize the association with cardiovascular events risk. Multiple testing may be required depending on the patient's condition and risk factors. It is important to educate individuals with high Lp (a) levels [defined as ≥70 mg/dl(150 nmol/L), maybe lower for Chinese (52)]. to maintain a healthy lifestyle, intensive management of other risk factors (e.g body mass, blood pressure, blood glucose etc.), and add proprotein convertase subtilisin/kexin type 9 (PCSK9) inhibitors when necessary, as PCSK9 inhibitors have been confirmed to lower Lp (a) levels (52). However, specific Lp (a)-lowering drugs are not available. Therefore, further studies are needed to investigate these interventions and evaluate the clinical benefits, to guide clinical practice for high Lp (a) levels in ACS patients. By decreasing high Lp (a) levels, it is possible to further reduce the risk of MACE and mortality, thus alleviating the disease burden worldwide. This meta-analysis represents a groundbreaking investigation, confirming Lp (a)'s prognostic significance in predicting MACE and all-cause mortality among ACS patients. However, some evidence demonstrates that cathepsin s, soluble LOX-1, and LDL-electronegativity, which have been implicated in the pathogenesis of atherosclerotic cardiovascular disease, have been related with prognosis in patients with ACS (54–56). All of these biomarkers, elevated during ACS, not measured and analysed in the included studies, could be associated with worse outcomes in ACS patients, may limit the interpretation of results. Additionally, several considerations merit attention. Firstly, the absence of individual-level data may introduce variability in pooled outcomes based on patient characteristics. Secondly, diverse cutoff values for elevated blood Lp (a) levels across studies hinder the establishment of a standardized threshold for Lp (a) elevation. Thirdly, the heterogeneity in pooled outcomes may stem from ACS subgroup, study design, follow-up duration, Lp (a) measurement methods, and cutoff values.The prognostic role of Lp (a) using continuous data couldn't be assessed due to insufficient information. Additionally, the exclusion of randomized controlled trials was necessary due to data limitations. Moreover, besides cathepsin s, soluble LOX-1, and LDL-electronegativity, the ACS, as acute inflammation, has potential to increase the level of Lp (a) and thus affect its measurement. Considering the potential confounders and above limitations, results of the present pooled analysis should be interpreted with caution. In spite of several limitations of our study, we confirmed that an elevated Lp (a) level is an independent predictor of MACE and all-cause mortality in ACS patients. The funnel plots and Egger's test examination revealed no significant publication bias, which makes our results more robust. Our evidence-based analysis validated previous studies reporting the different MACE outcomes in high level Lp (a) vs. low level patients with ACS (21, 25, 28, 36–38). More well-designed, large-scale prospective randomized studies with long-term follow-up are needed to further compare the MACE, all-cause mortality in high level vs. low level of Lp (a) patients with ACS.

## Conclusions

This meta-analysis underscores that elevated Lp (a) levels independently predict MACE and all-cause mortality in ACS patients. Because of minimal influence from lifestyle, diet, and medical interventions, Lp (a) measurement has the potential to enhance risk stratification in ACS. Future well-designed studies are crucial to investigate variations in Lp (a) prognostic significance among ACS subgroups. Furthermore, these investigations should conclusively establish Lp (a)'s role in refining risk stratification and modifying residual risk in ACS-diagnosed individuals.

## Data Availability

The original contributions presented in the study are included in the article/**Supplementary Material**, further inquiries can be directed to the corresponding author.
