# Supplementary material for: Prognostic value of elevated lipoprotein (a) in patients with acute coronary syndromes: a systematic review and meta-analysis
Source: Front Cardiovasc Med. 2024 May 9;11:1362893. doi: 10.3389/fcvm.2024.1362893 (PMC11112025; doi:10.3389/fcvm.2024.1362893)
Supplement: Supplementary file 2 [file Datasheet2.docx]

| Supplementary Table 2. Detailed search strategy in three databases. | |
| --- | --- |
| Database | Search strategy |
| Pubmed | (((("Angina, Unstable"[Mesh]) OR (((((((((((((((((((Angina, Unstable) OR (Anginas, Unstable)) OR (Unstable Anginas)) OR (Angina Pectoris, Unstable)) OR (Angina Pectori, Unstable)) OR (Unstable Angina Pectori)) OR (Unstable Angina Pectoris)) OR (Unstable Angina)) OR (Angina at Rest)) OR (Angina, Preinfarction)) OR (Anginas, Preinfarction)) OR (Preinfarction Angina)) OR (Preinfarction Anginas)) OR (Myocardial Preinfarction Syndrome)) OR (Myocardial Preinfarction Syndromes)) OR (Preinfarction Syndrome, Myocardial)) OR (Preinfarction Syndromes, Myocardial)) OR (Syndrome, Myocardial Preinfarction)) OR (Syndromes, Myocardial Preinfarction))) OR (("Myocardial Infarction"[Mesh]) OR ((((((((((((((Myocardial Infarction) OR (Infarction, Myocardial)) OR (Infarctions, Myocardial)) OR (Myocardial Infarctions)) OR (Cardiovascular Stroke)) OR (Cardiovascular Strokes)) OR (Stroke, Cardiovascular)) OR (Strokes, Cardiovascular)) OR (Myocardial Infarct)) OR (Infarct, Myocardial)) OR (Infarcts, Myocardial)) OR (Myocardial Infarcts)) OR (Heart Attack)) OR (Heart Attacks)))) OR (("Acute Coronary Syndrome"[Mesh]) OR ((((((Acute Coronary Syndrome) OR (Acute Coronary Syndromes)) OR (Coronary Syndrome, Acute)) OR (Coronary Syndromes, Acute)) OR (Syndrome, Acute Coronary)) OR (Syndromes, Acute Coronary)))) AND (((((" Lipoprotein(a) ") OR (" Lipoprotein Lp(a) ")) OR ("Lipoprotein (a) ")) OR ("Lipoprotein a ")) OR ("Lipoprotein (a-)")) |
| Embase^*^ | 1 'angina, unstable' OR 'anginas, unstable' OR 'unstable anginas' OR 'angina pectoris, unstable' OR 'angina pectori, unstable' OR 'unstable angina pectori' OR 'unstable angina pectoris' OR 'unstable angina' OR 'angina at rest' OR 'angina, preinfarction' OR 'anginas, preinfarction' OR 'preinfarction angina' OR 'preinfarction anginas' OR 'myocardial preinfarction syndrome' OR 'myocardial preinfarction syndromes' OR 'preinfarction syndrome, myocardial' OR 'preinfarction syndromes, myocardial' OR 'syndrome, myocardial preinfarction' OR 'syndromes, myocardial preinfarction'  2 'myocardial infarction' OR 'infarction, myocardial' OR 'infarctions, myocardial' OR 'myocardial infarctions' OR 'cardiovascular stroke' OR 'cardiovascular strokes' OR 'stroke, cardiovascular' OR 'strokes, cardiovascular' OR 'myocardial infarct' OR 'infarct, myocardial' OR 'infarcts, myocardial' OR 'myocardial infarcts' OR 'heart attack' OR 'heart attacks'  3 'acute coronary syndrome' OR 'acute coronary syndromes' OR 'coronary syndrome, acute' OR 'coronary syndromes, acute' OR 'syndrome, acute coronary' OR 'syndromes, acute coronary'  4 #1 OR #2 OR #3  5 'lipoprotein(a)'/exp OR 'lipoprotein(a)' OR 'lipoprotein lp(a)' OR 'lipoprotein (a)'/exp OR 'lipoprotein (a)' OR 'lipoprotein a'/exp OR 'lipoprotein a' OR 'lipoprotein (a-)'/exp OR 'lipoprotein (a-)'  6 #4 AND #5 |
| cochrane | ((((Angina, Unstable) OR (((((((((((((((((((Angina, Unstable) OR (Anginas, Unstable)) OR (Unstable Anginas)) OR (Angina Pectoris, Unstable)) OR (Angina Pectori, Unstable)) OR (Unstable Angina Pectori)) OR (Unstable Angina Pectoris)) OR (Unstable Angina)) OR (Angina at Rest)) OR (Angina, Preinfarction)) OR (Anginas, Preinfarction)) OR (Preinfarction Angina)) OR (Preinfarction Anginas)) OR (Myocardial Preinfarction Syndrome)) OR (Myocardial Preinfarction Syndromes)) OR (Preinfarction Syndrome, Myocardial)) OR (Preinfarction Syndromes, Myocardial)) OR (Syndrome, Myocardial Preinfarction)) OR (Syndromes, Myocardial Preinfarction))) OR ((Myocardial Infarction) OR ((((((((((((((Myocardial Infarction) OR (Infarction, Myocardial)) OR (Infarctions, Myocardial)) OR (Myocardial Infarctions)) OR (Cardiovascular Stroke)) OR (Cardiovascular Strokes)) OR (Stroke, Cardiovascular)) OR (Strokes, Cardiovascular)) OR (Myocardial Infarct)) OR (Infarct, Myocardial)) OR (Infarcts, Myocardial)) OR (Myocardial Infarcts)) OR (Heart Attack)) OR (Heart Attacks)))) OR ((Acute Coronary Syndrome) OR ((((((Acute Coronary Syndrome) OR (Acute Coronary Syndromes)) OR (Coronary Syndrome, Acute)) OR (Coronary Syndromes, Acute)) OR (Syndrome, Acute Coronary)) OR (Syndromes, Acute Coronary)))) AND (((((" Lipoprotein(a) ") OR (" Lipoprotein Lp(a) ")) OR ("Lipoprotein (a) ")) OR ("Lipoprotein a ")) OR ("Lipoprotein (a-)")) |
| ^*^ We retrieved articles from Embase via the Ovid (https://ovidsp.ovid.com/). | |
