# Supplementary material for: Prognostic value of elevated lipoprotein (a) in patients with acute coronary syndromes: a systematic review and meta-analysis
Source: Front Cardiovasc Med. 2024 May 9;11:1362893. doi: 10.3389/fcvm.2024.1362893 (PMC11112025; doi:10.3389/fcvm.2024.1362893)
Supplement: Supplementary file 3 [file Datasheet3.docx]

| Supplementary Table S3. Quality evaluation of the eligible studies with Newcastle–Ottawa scale. | | | | | | | | | |
| --- | --- | --- | --- | --- | --- | --- | --- | --- | --- |
| Study | Selection | | | | Comparability | | Outcome | | |
|  | Representative-ness | Selection of  non-exposed | Ascertainment  of exposure | Outcome not present at start | Comparability on most important factors | Comparability on other risk factors | Assessment of outcome | Long enough follow-up (median≥1 year) | Adequacy  (completeness) of follow-up |
| Kallmeyer, A et al.（21） | * | * | * | * | - | - | * | * | * |
| Hoang,S et al.（23） | * | * | * | * | - | - | * | - | * |
| Xue Y, et al.（34） | * | * | * | * | - | - | * | * | * |
| Park J,et al.（35） | * | * | * | * | - | - | * | * | * |
| Zhu L,et al.（36） | * | * | * | * | - | * | * | * | * |
| Yang,S,et al.（22） | * | * | * | * | * | * | * | - | * |
| Wang,Y,et al.（25） | * | * | * | * | - | - | * | * | * |
| Dai,K,et al.（37） | * | * | - | * | * | * | * | * | - |
| Takahashi,D,et al.（38） | * | * | * | * | - | - | * | * | * |
| Sang,T,et al.（26） | - | - | * | * | * | - | * | * | * |
| Cui,C,Y,et al.（39） | * | * | * | * | * | - | * | * | * |
| Mitsuda, T.et al2015.（27） | * | * | * | * | - | - | * | * | * |
| Roth,C,et al.（28） | * | * | * | * | * | - | * | * | * |
| Gómez,M,et al.（20） | * | * | * | * | - | - | * | - | * |
| Mitsuda,T,et al2019.（29） | * | * | * | * | * | - | * | * | * |
| Gencer,B,et al.（40） | * | * | * | * | * | * | * | * | * |
| Li,Q,et al.（41） | - | - | * | * | - | - | * | * | * |
| Wohlfahrt,P,et al.（42） | * | * | * | * | * | - | * | * | * |
| *indicates criterion met; - indicates significant of criterion not met. | | | | | | | | | |
|  | | | | | | | | | |
